# A Tree Crown Segmentation Approach for Unmanned Aerial Vehicle Remote Sensing Images on Field Programmable Gate Array (FPGA) Neural Network Accelerator

**DOI:** 10.3390/s25092729

**Published:** 2025-04-25

**Authors:** Jiayi Ma, Lingxiao Yan, Baozhe Chen, Li Zhang

**Affiliations:** 1 College of Science, Beijing Forestry University, Beijing 100091, China; jiayima@bjfu.edu.cn (J.M.); baozhechen183@bjfu.edu.cn (B.C.); 2 Tsinghua Shenzhen International Graduate School, Tsinghua University, Shenzhen 518071, China; yanlingxiao4869@126.com

**Keywords:** tree crown segmentation, neural network accelerator, SoC design

## Abstract

Tree crown detection of high-resolution UAV forest remote sensing images using computer technology has been widely performed in the last ten years. In forest resource inventory management based on remote sensing data, crown detection is the most important and essential part. Deep learning technology has achieved good results in tree crown segmentation and species classification, but relying on high-performance computing platforms, edge calculation, and real-time processing cannot be realized. In this thesis, the UAV images of coniferous Pinus tabuliformis and broad-leaved Salix matsudana collected by Jingyue Ecological Forest Farm in Changping District, Beijing, are used as datasets, and a lightweight neural network U-Net-Light based on U-Net and VGG16 is designed and trained. At the same time, the IP core and SoC architecture of the neural network accelerator are designed and implemented on the Xilinx ZYNQ 7100 SoC platform. The results show that U-Net-light only uses 1.56 MB parameters to classify and segment the crown images of double tree species, and the accuracy rate reaches 85%. The designed SoC architecture and accelerator IP core achieved 31 times the speedup of the ZYNQ hard core, and 1.3 times the speedup compared with the high-end CPU (Intel CoreTM i9-10900K). The hardware resource overhead is less than 20% of the total deployment platform, and the total on-chip power consumption is 2.127 W. Shorter prediction time and higher energy consumption ratio prove the effectiveness and rationality of architecture design and IP development. This work departs from conventional canopy segmentation methods that rely heavily on ground-based high-performance computing. Instead, it proposes a lightweight neural network model deployed on FPGA for real-time inference on unmanned aerial vehicles (UAVs), thereby significantly lowering both latency and system resource consumption. The proposed approach demonstrates a certain degree of innovation and provides meaningful references for the automation and intelligent development of forest resource monitoring and precision agriculture.

## 1. Introduction

The application of automatic tree crown detection in high-resolution forest remote sensing images has been a topic attracting extensive attention over the last ten years [1,2,3,4]. Tree crown detection is the utmost and essential part of almost all methods of forest resource inventory and management based on remote sensing data [5,6]. A lot of work on forest resource inventory and crown delineation by using remote sensing data has been proposed, which has achieved good results in both the individual level and classification accuracy [7]. Among these methods, tree crown detection is an indispensable key step, and its results play a decisive role in research. However, window-based filters are used in much of the work to find local maxima as potential tree crowns. It is theoretically feasible, but the implementation might fall short of expectations. A suitable filter size for various tree crowns is usually difficult to find, even if the adaptive filter method is adopted [8].

Recently, convolutional neural networks using deep learning technology have been applied in image classification [9,10], target detection [11,12], image segmentation, etc. [13]. Different from the traditional method of using filters for feature determination, the application of the convolution neural network performs better in learning advanced semantic information from training samples. In the aspect of tree crown detection, the convolutional neural network performs better in different scenes than the adaptive filters as the accuracy of supervised machine learning is usually high [14].

With the constant increase in the data amount and collection speed, more remote sensing data processing work based on board has been put forward [15]. In order to eliminate the delay between data collection and processing as much as possible and also to reduce the amount of data transmitted from satellites to the ground, FPGA has become one of the most popular on-board remote sensing data processing platforms among many high-performance platforms [16] and graphics processors [17]. The reasons are as follows: Firstly, compared with high-performance platforms and graphics processors, FPGA is usually smaller in size and weight and has the inherent reprogramming ability. Secondly, compared with graphics processors, FPGA consumes less power and generates less heat due to its characteristics for hardware programming [18]. Apart from that, FPGA has the advantages of parallel computing and low delay in the optimization of neural networks. After being accelerated by FPGA hardware, the neural network algorithm can obtain faster computation speed and lower power consumption than on MCU or PC by controlling multiple accelerators through the assembly line to conduct computation, reuse RAM resources, make floating-point number hardware-oriented to reduce the number of instructions, and reduce the operation accuracy [19,20].

Therefore, FPGA is widely used in remote sensing detection. In hyperspectral remote sensing, since more hyperspectral [21] applications require real- or near-real-time processing capabilities, reconfigurable hardware solutions such as field-programmable gate arrays have been consolidated during the last years as one of the standard choices for the fast processing of hyperspectral remotely sensed image [22]. Due to the power limitation of (on-board space platforms) (spaceborne or airborne), the ultra-low power consumption of FPGA is widely welcomed by researchers [23]. In some scenes with high demand for real-time processing and computing performance, such as natural disaster prediction, one of the solutions is to process the data using FPGA [23]. With the increasing complexity of remote sensing image analysis algorithms, researchers will also tend to use FPGA for hardware acceleration to pursue higher computing performance [24].

In recent years, scientists have applied neural network to the tree crown detection [25,26,27]. In 2018, Ramesh Kestur et al. innovatively integrated the Extreme Learning Machine (ELM), a single-hidden-layer feedforward neural network classifier, with spectral–spatial analysis. Their study confirmed that ELM outperformed traditional K-means spectral–spatial clustering in classification accuracy, leveraging its computational efficiency (suitable for real-time processing) and synergistic optimization of spectral–spatial features. However, this method required post-processing spatial filtering to correct the misclassifications and manual adjustment of geometric thresholds (e.g., area and eccentricity), indicating limitations in automation [17]. In 2019, Ben G. Weinstein et al. proposed a semi-supervised deep learning framework for RGB-based tree crown detection. By generating initial training samples through unsupervised LiDAR detection, this approach effectively reduced reliance on manual annotations. While cross-modal learning between LiDAR and RGB data enhanced model generalization, its performance was constrained by LiDAR dependency and exhibited reduced accuracy in dense canopy environments [28]. In 2020, Jose R. G. Braga et al. achieved breakthrough results by applying Mask R-CNN to tropical forest crown detection. This method demonstrated robust instance segmentation capabilities for heterogeneous canopy structures and improved sample diversity through synthetic data augmentation. Nevertheless, it incurred high computational costs, potential domain discrepancies between synthetic and real-world data, and time-intensive training processes [27]. Ben G. Weinstein et al. extend a recently developed deep learning approach to include data from a range of forest types and explore the potential for building a universal tree detection algorithm [28]. Alin-Ionut Plesoianu et al. developed a reproducible deep learning ensemble based on Single Shot MultiBox Detector (SSD) models for individual tree crown (ITC) detection and species classification. Although the ensemble enhanced detection stability through multi-network integration, performance plateaued beyond two submodels, and its lack of spatial feature integration resulted in inferior crown boundary delineation compared to segmentation-based approaches [5]. In 2021, Zhenbang Hao et al. explored training a mask region-based convolutional neural network (Mask R-CNN) for automatically and concurrently detecting discontinuous tree crowns and height of Chinese fir (Cunninghamia lanceolata (Lamb) Hook) in a plantation. Their framework achieved pixel-level instance segmentation and multi-attribute prediction, excelling in complex backgrounds and young stands. However, the intricate architecture increased computational demands, while reliance on high-precision instance masks and height labels elevated annotation costs [29]. Zhafri Roslan et al. proposed a noise-canceling GAN-based Bodel by averaging the weights of a compressed image and a non-compressed image. Combined with the RetinaNet single-stage detector, this method improved small-target detection but was limited to crown localization without precise contour extraction, exhibiting weaker generalization capabilities compared to ensemble or segmentation models [30]. In 2023, Yang Liu et al. significantly enhanced the accuracy and boundary consistency of canopy detection by integrating the strengths of deep convolutional neural networks (DCNNs) with decision trees and introducing superpixel segmentation technology. This approach facilitated direct mapping from pixels to semantic labels, thereby reducing the necessity for post-processing. However, the model’s performance is notably influenced by hyperparameters such as learning rate, tree depth, and the number of trees, necessitating careful tuning. Additionally, it heavily depends on large volumes of annotated data and exhibits suboptimal performance in segmenting individual trees within high-density canopy areas [31].

Among segmentation architectures, U-Net has emerged as a benchmark model due to its unique encoder–decoder structure with skip connections, making it effective in capturing spatial detail across different scales. Originally proposed for biomedical image segmentation, U-Net’s ability to handle limited training data through data augmentation and its effectiveness in capturing fine-grained boundaries have led to adaptations in remote sensing applications. Recent variants like Sharp U-Net further optimize this architecture through depthwise separable convolutions, demonstrating 12.7% higher boundary accuracy in complex segmentation tasks compared to standard implementations [32]. However, the direct application of U-Net in tree crown segmentation faces challenges including excessive parameter counts and computational complexity, which hinder real-time deployment on edge devices.

In the existing research, the application of FPGA platforms in canopy remote sensing image processing remains relatively limited. In 2019, the team led by Weijia Li at Tsinghua University proposed the PF-TCD algorithm [33], which achieved the real-time detection of oil palm canopies from Quickbird satellite imagery in southern Malaysia. However, their focus was on traditional image processing and did not involve neural network acceleration. The current mainstream direction is to integrate computer vision and electronic technologies to promote intelligent forestry management, yet there is no literature reported on FPGA-based neural network acceleration for canopy segmentation. Therefore, it can be concluded that this research topic possesses a certain degree of cutting-edge and innovative characteristics, providing a solution with both academic value and application potential for the automated monitoring of forest resources.

## 2. Data

**Figure 1:**
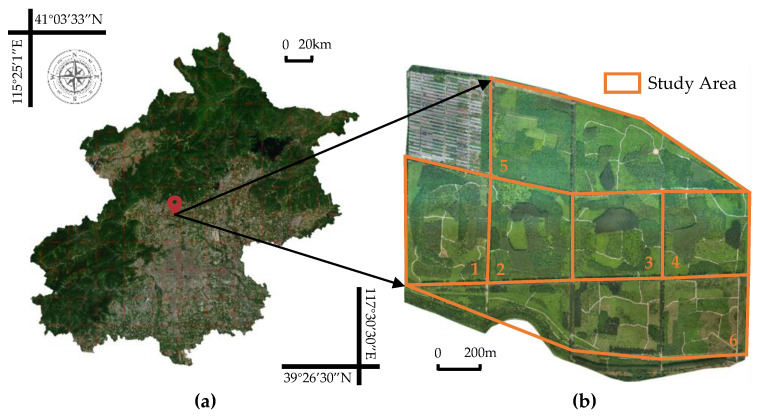
(**a**) Geographical location of the study area; (**b**) remote sensing image of UAV (1 to 6 in the figure are the study areas).

The study site is situated within the Jingyue Ecological Forest Farm, located in Changping District, Beijing, at a latitude and longitude of 40°10′52″ N, 116°11′24″ E (Figure 1). The local climate is characterized by a typical temperate semi-humid and semi-arid monsoon climate, with an average annual temperature of approximately 19 °C, humidity of 60% R.H., and rainfall of 600 mm. These climatic features are highly consistent with the ecological forest areas of the North China urban agglomeration, demonstrating significant regional representativeness. The terrain is a relatively flat plain with undulations of less than 2 m, and the average elevation of the study area is about 43 m. Such topographic conditions are conducive to the stable acquisition of drone aerial data and avoid interference from mountain shadows in remote sensing image analysis.

**Figure 2:**
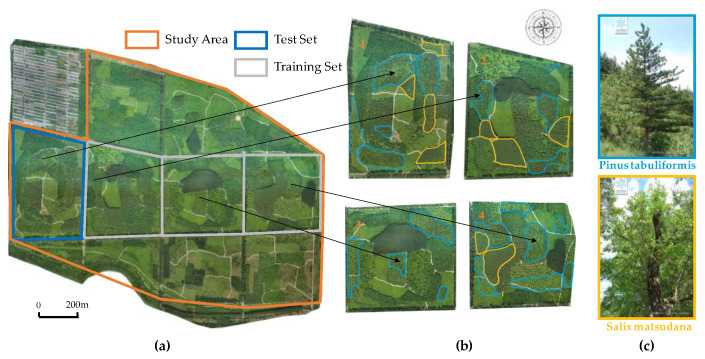
(**a**) Sample area of the training set and test set (area 1: test set; areas 2, 3 and 4: training set); (**b**) distributions of the studied tree species; (**c**) pictures and names of the studied tree species.

According to the division of the forest farm and the direction of the road in the study area, it is divided into six regions. The data sources for this study are primarily concentrated in the four continuous regions located in the central quadrangle of the forest farm, as depicted in Figure 1b, which are numbered 1, 2, 3, and 4, respectively. The research area consists of a manmade forest that is accessible to society, characterized by a blocky mixed forest composed of various coniferous and broad-leaved trees. In this area, coniferous trees are relatively sparse while broad-leaved trees are relatively dense. There are seven species of conifers present throughout the entire area including Pinus tabuliformis, Ginkgo biloba, and Juniperus chinensis. Additionally, there are four types of broad-leaved trees: Styphnolobium japonicum, Salix matsudana, Ailanthus altissima, and Populus nigra. Due to time constraints on research activities, it was not feasible to complete calibration for all the tree species datasets. Therefore, one of the most widely distributed coniferous broad-leaved trees—namely Pinus tabuliformis and Salix matsudana—has been selected to ensure representativeness in this study. The distribution patterns for tree species can be observed in Figure 2b, with corresponding images provided in Figure 2c.

The orthophoto image of the forest area utilized was acquired from the unmanned aerial vehicle (UAV) orthophoto imagery employed in Chong Zhang et al.’s study [16]. The UAV aerial images were taken on the morning of 26 May 2023, under subdued sunlight and gentle wind conditions, which were conducive to remote sensing image data collection.

Based on the orthophoto image of the entire forest area, the selected study area is divided. As depicted in Figure 2a, images from plots 2 to 4 are utilized for creating a training set and validation set, while the test set is located in an independent plot 1, avoiding geographical overlap with the training set to reduce the impact of spatial autocorrelation. Within plots 2, 3, and 4, images with a resolution of 512 pixels are segmented into 100 segments with a length–width ratio of 1:1. Among these segments, there are equal numbers (50 each) containing Pinus tabuliformis forests and Populus euphratica forests. The training images are further partitioned into a training set and validation set at a ratio of 7:3. Similarly, within plot number one, an additional hundred test images with the same resolution exclusively feature the Pinus tabuliformis and Populus euphratica tree species.

The deep learning model utilized in this study is supervised learning, necessitating the manual annotation of input images prior to model training [17]. The Labelme annotation tool, based on the Anaconda3 virtual environment, was employed for classifying and annotating the segmentation of tree crown amplitude in images. Using the polygon creation tool, we manually delineated the canopy boundaries of each tree on the RGB images, assigned corresponding tree labels based on species information, and created a standard dataset for canopy segmentation in accordance with the PASCAL-VOC2012 dataset format.

## 3. Methods

### 3.1. U-Net-Light

#### 3.1.1. U-Net Semantic Segmentation Model

U-Net is a classic semantic segmentation model proposed by Ronneberger et al. at the MICCAI 2015 conference [28,34]. U-Net model training can be based on small datasets composed of hundreds of images. For drone canopy remote sensing images, obtaining and creating a large dataset is also difficult, and U-Net solves this problem very well.

**Figure 3:**
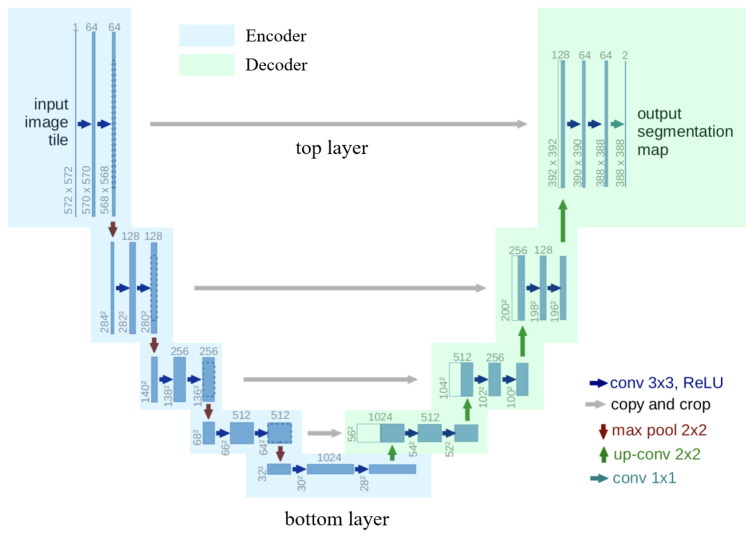
Structure diagram of classic U-Net.

U-Net employs a decoder–encoder architecture, as illustrated in Figure 3, featuring a U-shaped structure on the left side representing the encoder component. This encoder, denoted as ‘Encoder’, comprises layers with a blue background that signify the feature extraction process. The encoder primarily consists of convolutional layers and max pooling layers. The convolutional layers utilize a 3 × 3-pixel convolution kernel with a stride of 1 pixel. As no edge padding is applied, the image size decreases by two pixels following convolution. The max pooling layer, with a kernel size of 2 × 2 pixels, reduces both the image length and width by half, thereby achieving downsampling. The feature map generated after convolution is preserved prior to each downsampling operation. Notably, the number of feature maps produced by each convolutional layer after the max pooling layer exceeds that of the previous convolution by one, as depicted in Figure 3. The number of feature maps in the convolutional layers, progressing from the top to the bottom of the U-shaped structure, are 64, 128, 256, 512, and 1024.

The decoder of the U-Net is located on the right side of the U-shaped structure, consisting of layers with the background color green in Figure 3, mainly including convolutional layers, feature fusion layers, and upsampling layers. The decoder’s convolutional layers are the same as those in the encoder, except for the last convolutional layer’s kernel size of 1 × 1 (pixel) size, which is not described in detail. The feature fusion layer is used to stack the feature maps retained before each downsampling and the feature maps upsampled to completion along the channel dimension, with the same dimension. The upsampling process is the opposite of the downsampling process, achieving the effect of enlarging the feature map, enlarging the feature map to twice the original length and width, corresponding to the downsampling dimension, and forming the U-shaped structure.

#### 3.1.2. VGG16 Backbone Network

**Figure 4:**
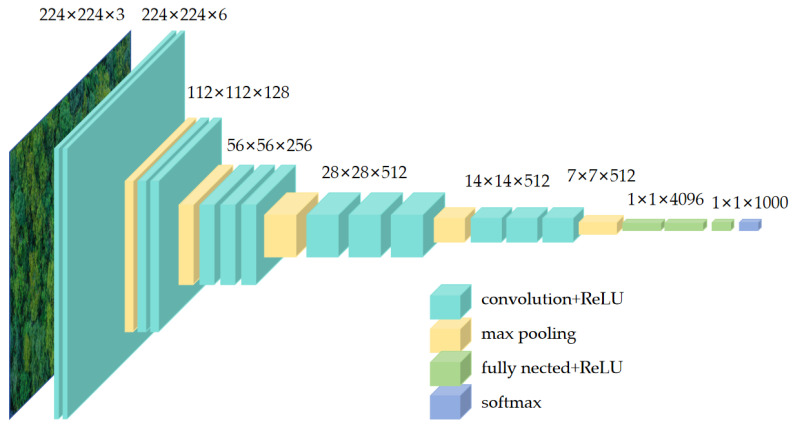
Structure diagram of VGG16.

VGG16 (Visual Geometry Group) is a deep convolutional neural network architecture proposed by the Visual Geometry Group in 2014 [35]. VGG networks are widely used in computer vision tasks such as image classification, object detection, and semantic segmentation, and the simplicity and ease of implementation of its network structure have made VGG one of the classic models in the field of deep learning. VGG16 network uses continuous small convolutional kernels (3 × 3) and pooling layers to build a deep neural network, and the feature extraction part of the network has a depth of 16 layers. The network architecture of VGG16 is composed of alternating convolutional and pooling layers stacked together, and the final classification is performed using a fully connected layer. The network structure diagram is shown in Figure 4.

The backbone network of VGG16 is the convolutional layer and pooling layer in front of the fully connected layer, which mainly plays the role of feature extraction, and the function of the U-Net decoder is the same.

#### 3.1.3. The Improved Model U-Net-Light

The U-Net network is one of the classical networks of semantic segmentation and belongs to the convolutional neural network. For the parameter Np of each layer of the convolutional neural network, there is the following formula:

(1) $$N_{p}=\;C_{o}\times (C_{i}\times K_{w}\times K_{h}+1)$$

In the formula, $C_{o}$ represents the number of channels in the output feature graph, which is also the number of convolution kernels; $C_{i}$ denotes the number of channels in the input feature map; $K_{h}$ signifies the height of the convolution kernel; and $K_{w}$ indicates the width of the convolution kernel.

**Figure 5:**
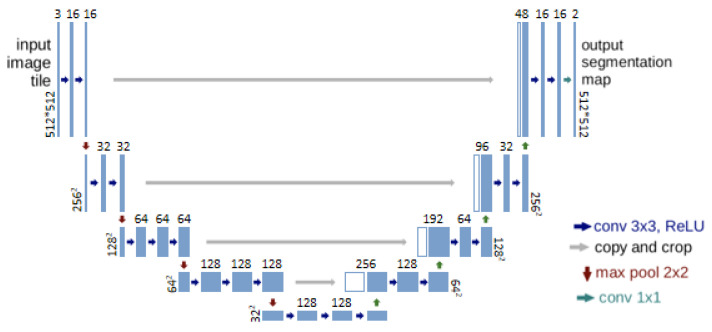
Structure diagram of U-Net-light.

Based on the original network structure of U-Net, it can be estimated that the number of parameters is over 25 MB. For the network model deployed on the edge end, the excessively large number of parameters affects the computing efficiency; at the same time, the problem of shrinking the feature map size affects the alignment of storage addresses and increases the deployment difficulty of FPGA. Therefore, under the premise of ensuring the prediction effect, in order to reduce the number of parameters, lower the deployment difficulty, and adapt to the multi-species research of remote sensing images, this paper proposes the following improvement scheme and designs the U-Net-light model based on the VGG16 main body network for optimization and lightweighting. The model structure is shown in Figure 5.

In selecting the backbone network, this paper adopts the classic VGG16 as the feature extraction module, replacing the decoder structure in the original U-Net. Compared to more complex backbone networks such as ResNet and DenseNet, VGG16 features a concise structure that is deployment-friendly, making it particularly suitable for applications on embedded devices or edge platforms. Its consecutive 3 × 3 convolutional structure performs stably in capturing image details, while the network’s clear layering and controllable parameter distribution facilitate engineering optimization and model compression. Although ResNet excels in image classification tasks through the use of residual connections, its original design is more suited for global feature extraction rather than the detail restoration tasks required by the U-Net decoder. While residual connections can alleviate the vanishing gradient problem, they also increase model complexity and complicate FPGA deployment. For instance, ResNet-50 has approximately 25.6 M parameters, which does not align with the objective of lightweight design. The Inception series of networks is renowned for its multi-branch structures, which are capable of capturing multi-scale features; however, they exhibit significant disadvantages regarding computational complexity and parameter count. For instance, the FLOPS of Inception V3 are typically higher than those of VGG16, imposing greater demands on the computational capabilities of FPGAs. Additionally, their intricate multi-branch designs increase the difficulty of model implementation and hinder the goal of lightweight design. In contrast, MobileNet achieves lightweight architecture through depthwise separable convolution, resulting in a relatively small number of parameters (e.g., MobileNet V1 has approximately 4.2 M parameters), making it seemingly suitable for edge deployment. However, this architecture compromises feature extraction capabilities, particularly in tasks such as canopy segmentation that require high-edge clarity and spatial detail, often leading to blurred boundaries and inaccurate localization. Considering these factors, VGG16 offers a more favorable balance between semantic expression capability, computational complexity, and deployment feasibility, and is, therefore, selected as the backbone network structure for the lightweight model in this study.

1. Model Lightweight

Based on the original structure of U-Net, the U-Net-light model reduces the number of convolutional feature graph channels to 1/4 of the original one. According to Formula (1), the number of parameters at this time decreases to 1/16 of the original, that is, 1.81 MB. At the same time, the backbone of VGG16 is used to replace the original decoder structure, and the extraction ability of image feature information is improved by increasing the number of convolutional layers, and the impact of the reduction in the number of feature maps on the extraction of deep semantic information is reduced. In addition, the backbone structure of VGG16 is used to split a feature graph with 256 channels into two feature graphs with 128 channels at the bottom of the U-shaped network model, and the number of parameters is further reduced to 1.56 MB.

2. Feature Map Length and Width Standardization

The U-Net-light model uses the filling strategy in all the convolution layers. Before the convolution operation, 0 values are filled in the edge of the feature graph to ensure that the length and width of the feature graph after convolution remain unchanged. At the same time, the size of the input image is fixed to 256 pixels (or 128 or 64) to ensure that it is an integer multiple of 8, which is convenient for byte alignment when storing the feature map.

3. Multispecies Classification

The input image format is changed from grayscale to RGB three-color 24-bit color map to retain more information about remote sensing images. The Softmax activation function is used instead of the Sigmoid activation function to output the segmentation results of multiple tree species [15], which is suitable for the classification scenario of an artificial block mixed forest in a forest farm.

### 3.2. Network Construction Based on PyTorch and ONNX

#### 3.2.1. Network Training

In this paper, we used the self-constructed dataset to complete the training of the neural network and realized the crown segmentation [28,36] of Pinus tabuliformis and Salix matsudana. GPU was used to complete the training process, and the computing platform was NVIDIA GeForce RTX 3080 Ti (NVIDIA, Santa Clara, CA, USA). The specifications are shown in Table 1.

The Adaptive Moment Estimation algorithm is used for U-Net-light training, and the feature extraction network is VGG16. The training environment is the Python virtual environment created in Anaconda3 with Python version 3.8.8. PyTorch framework (version 1.7.1) is used to build the model and CUDA 11.0 is adapted. The training setting parameters are shown in Table 2.

#### 3.2.2. Model Transformation

After the PyTorch model is trained, the parameter file of the model is stored in. pth format, which must be opened under the PyTorch framework to read, and the weight value cannot be directly exported. Therefore, this paper studies the conversion of the trained PyTorch model into the ONNX model using the function package ONNX 1.12.0. ONNX is an open source format for machine learning models that supports interoperability across framework models. At the same time, ONNX can be visualized with the help of Netron’s online website to facilitate the export of model parameters and the construction of the calculation process.

#### 3.2.3. Evaluation Index

In order to study the inference effect of neural network model on tree classification and crown width segmentation, pixel accuracy rate (PA), class pixel accuracy rate (CPA), class average pixel accuracy rate (MPA), intersection ratio (IoU), and average intersection ratio (MIoU) were used as the quantitative evaluation indices of semantic segmentation.

(2) $$PA=\frac{T_{P}+T_{N}}{T_{P}+T_{N}+F_{P}+F_{N}}$$

(3) $$P_{i}=\frac{T_{P}}{T_{P}+F_{P}},i=1,2,\ldots ,n$$

(4) $$MPA=\frac{\sum_{i=1}^{n}P_{i}}{n}$$

(5) $$IoU_{i}=\frac{T_{P}}{T_{P}+F_{P}+F_{N}},i=1,2,\ldots ,n$$

(6) $$MIoU=\frac{\sum_{i=1}^{n}IoU_{i}}{n}$$

In the given formula, n signifies the number of classifications, which in this study is 3, and $T_{P}$ stands for the number of pixels that are accurately identified as a specific tree species. $T_{N}$ refers to the number of pixels that correctly identify other tree species, excluding the particular tree species in question. $F_{P}$ denotes the number of pixels that are incorrectly identified as a tree species, while $F_{N}$ represents the number of pixels that falsely classify a tree species as another.

### 3.3. C++ Algorithm Implementation

The neural network built using Python language has been trained and verified through testing, proving that the network has achieved the expected function. The next step is code migration. Neural network models designed directly by high-level programming languages (such as Python) cannot be directly deployed to platforms such as FPGA. Hardware description language (HDL) is usually used for RTL-level code writing and comprehensive burning to the FPGA platform. However, the development cycle of FPGA using HDL is long and difficult. Therefore, this study chooses the HLS development method, writes the RTL logic through C/C++, and realizes the RTL-level code directly and comprehensively. The tools provided by Xilinx, such as Vitis HLS, are used to realize the comprehensive conversion of C source code and the compatibility of FPGA hardware.

On the other hand, Python language built-in library functions can directly achieve multidimensional matrix addition and multiplication operations, while C/C++ does not have the tools to directly complete matrix operations, so it is necessary to use the underlying language to analyze and rewrite its model implementation process, comprehensive use of sequence structure, selection structure, and loop structure to complete process-oriented programming and to achieve core operational functions. Programming at the C/C++ level is designed to aid hardware design, further analyze functionality, and more efficiently target code refactoring for the forward inference process. Based on this, this paper implements three common algorithms of neural networks on the Visual Studio 2019 integrated development environment, including all the operators used in the U-Net-light model, and designs data structures, that can set specific values such as convolution kernel size, sliding step size, filling mode, input and output image resolution, etc., to improve the reusability of code modules.

#### 3.3.1. Two-Dimensional Convolution

Convolutional layers are the most numerous in the U-Net-light model, with 22 convolutional layers in the decoder and decoder. As shown in Algorithm 1, variable weights.filters represents the number of convolutional cores as well as the number of channels for output images. The variable convIn.channels represents the number of channels in the input image; fdim is the length and width of the convolution kernel. The stride is always 1, and the convolution kernel is always 1. In the U-Net-light model, when the fdim value of the last convolution layer is 1, the fdim value of the remaining convolution layers is constant 3, and the convolution kernel size can be regarded as constant 3, which is conducive to the architecture design of the later accelerator. When the convolution kernel size is 3, in order to keep the length and width values of the input and output images unchanged, the zero-value filling strategy is used to store the input feature map in the array convTmp, and the edge of the image is filled with 0 values during the stored procedure. Therefore, compared with the input feature map, the stored feature map in the array convTmp has 2 more pixels on the original length and width values of the image. Each time a convolution check image is used to complete a calculation, a new feature map is generated. The resolution of the input and output images is dim, which means convIn.height = convIn.width = dim.
**Algorithm 1** Convolution1:**procedure** <Convolution> (convIn, weights, bias);2:  stride ← 13:  OH ← ((convIn.height + 2 × padding − fdim)/stride) + 14:  OW ← ((convIn.width + 2 × padding − fdim)/stride) + 15:  **for** k: = 0 **to** (convIn.channels-1) **do**6:    **for** i: = 0 **to** (padding-1) **do**7:      **for** j: = 0 **to** (convIn.width + 2 × padding-1) **do**8:        convTmp(k, i, j) ← 09:        convTmp(k, j, i) ← 010:        convTmp(k, convIn.width + 2 × padding − 1 − i, j) ← 011:        convTmp(k, j, convIn.width + 2 × padding − 1 − i) ← 012:    **for** i: = padding **to** ((convIn.width + 2 × padding − 1)-padding) **do**13:      **for** j: = padding **to** ((convIn.width + 2 × padding − 1)-padding) **do**14:        array(k, i, j) ← input(k, i − padding, j − padding)15:  **for** i: = 0 **to** (weights.filters − 1) **do**16:    **for** x: = 0 **to** (h.dim − 1) **do**17:      **for** y: = 0 **to** (w.dim − 1) **do**18:        sum ← 019:        **for** j: = 0 **to** convIn.channels − 1 **do**20:          **for** k: = x **to** (x − 1 + fdim) **do**21:            **for** l: = y **to** (y − 1+ fdim) **do**22:              sum ← sum + convTmp(j, k, l) × weights(i, j, k − x, l − y)23:        output(i, x, y) ← sum + bias(i)24:  **return** convOut

#### 3.3.2. Maxpool

Algorithm 2 is the implementation of the Maxpool layer. In the network model of this study, the Maxpool layer plays the role of downsampling. The U-Net-light model has four Maxpool layers. fdim is always 2, and for each 2 × 2 pixel block, the maximum value is taken as the output result, so that the size of the input feature map can be reduced to the original 1/2 and output. Each channel of the feature map is independent of each other. Compared with the convolutional layer, the maximum pooling layer has simple operation and low data dependence, and only the CPU processor can achieve high-performance calculation.
**Algorithm 2** Maxpool1:**procedure** <Maxpool> (inputMap, fdim, stride)2:  hOut ← (inputMap.height − fdim)/stride + 13:  wOut ← (inputMap.width − fdim)/stride + 14:  **for** i: = 0 **to** (inputMap.channels-1) **do**5:    **for** j: = 0 **to** (hOut-1) **do**6:      **for** k: = 0 **to** (wOut-1) **do**7:        max ← Minimal negative value8:        **for** l: = j × s **to** (j × s + fdim-1) **do**9:          **for** m: = k × s **to** (k × s + fdim-1) **do**10:            point ← input(i, l, m)11:            if max < point then12:              max ←point13:        output(i, j, k) ← max14:**return** output

#### 3.3.3. Upsampling

Algorithm 3 is the implementation of the upper sampling layer, which uses a bilinear interpolation method with corner alignment, which is the inverse process of the average pooling layer. The bilinear interpolation method is used to ensure that the feature map is not distorted after sampling, and that the calculation amount is reduced without introducing more weight calculation.
**Algorithm 3** Upsampling1:**procedure** <Upsampling> (inputMap, sizeOut)2:  **for** channel: = 0 **to** (inputMap.channelfmap-1) **do**3:    **for** row: = 0 **to** (sizeOut-1) **dos**4:      **for** col: = 0 **to** (sizeOut-1) **do**5:        x ← row × (inputMap.sizeIn-1.0)/(sizeOut-1.0)6:        y ← col × (inputMap.sizeIn-1.0)/(sizeOut-1.0)7:        xi ← (int) x8:        yi ← (int) y9:        xa ← x − xi10:        ya ← y − yi11:        **if** (row < sizeOut-1 && col < sizeOut-1) **then**12:          outputMap[channel][row][col] ← inputMap[channel][xi][yi] × (1 − xa) × (1 − ya) + inputMap[channel][xi + 1][yi] × xa × (1 − ya) + inputMap[channel][xi][yi + 1] × (1 − xa) × ya + inputMap[channel][xi + 1][yi + 1] × xa × ya13:        **else if** (row == sizeOut-1 && col == sizeOut-1) **then**14:          outputMap[channel][row][col] = inputMap[channel][xi][yi]15:        **else if** (row == sizeOut-1) **then**16:          outputMap[channel][row][col] ← inputMap[channel][xi][yi] × (1 − ya) + inputMap[channel][xi][yi + 1] × ya17:        **else if** (col == sizeOut-1) **then**18:        outputMap[channel][row][col] ← inputMap[channel][xi][yi] × (1 − xa) + inputMap[channel][xi + 1][yi] × xa19:        **else then**20:          outputMap[channel][row][col] ← 021:  **return** outputMap

## 4. FPGA Implementation

**Figure 6:**
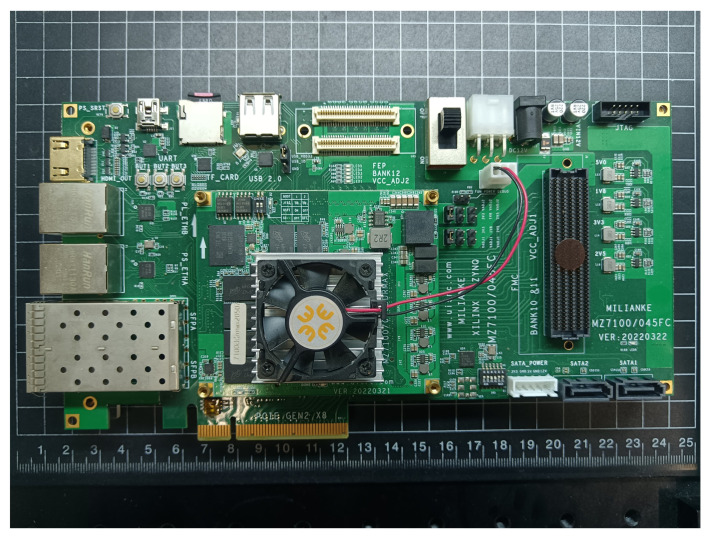
ZYNQ-MZ7100 development board.

The ZynQ-MZ7100 development board (MLK, Changzhou, Jiangsu Province, China) is utilized for the synthesis and implementation of the SoC design featuring a neural network accelerator, as depicted in Figure 6. The hardware specifications of the development board are detailed in Table 3. The semantic segmentation of tree crown images is achieved through a collaborative software and hardware approach utilizing the forward reasoning process of the neural network model. The selected hardware resources on the development board for this research are moderate, indicating that the proposed neural network accelerator design scheme can be adapted to other Xilinx series development platforms with a degree of universality. The programmable SoC model installed on the development board is XC7Z100FFG900-2I (Xilinx, SAN Jose, CA, USA), with the chip parameters listed in Table 3.

### 4.1. SoC Design

**Figure 7:**
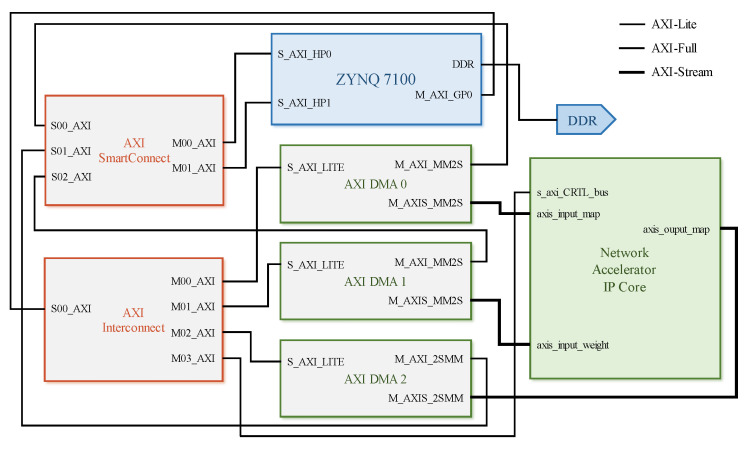
Topological graph about SoC design of neural network accelerator.

The system-on-chip (SoC) top-level design comprises four modules: The first module features an ARM processor core, with a single-core processor serving as the control core in this study. The second module is dedicated to the PL-end accelerator, focusing on enhancing the computational performance of specialized algorithmic components within the neural network that are not efficiently handled by the CPU, such as classical two-dimensional convolution operations. The third module consists of AXI DMA for the efficient transmission of feature maps and weights. Lastly, the fourth module encompasses the on-chip bus. This research design primarily utilizes the AXI4-Lite interface for configuring the control parameters and the DMA initialization of the neural network accelerator IP core, while employing AXI4-Stream and AXI-Full for data and weight transmission. Figure 7 illustrates the hardware topology of SoC design in this study, which integrates both software and hardware elements. Within this model, only two parameters need to be set in advance for the convolutional layer, with a significantly smaller number of offset values compared to weights. To facilitate acceleration processes, offset values are stored in BRAM with high reading and writing efficiency accessible by both the PL side and PS side within FPGA resources being relatively small. In the ZYNQ 7100 SoC (MLK, Changzhou, Jiangsu Province, China), utilized as the development platform in this study, the BRAM capacity is limited to only 26.5 Mb (3.3 MB), which restricts its ability to store weights for larger models such as Lenet-5 neural networks. To ensure design versatility, off-chip DDR at the PS end is employed for storing neural network weights. The ZYNQ PS terminal is utilized for controlling data stream transmission and configuring the control registers of each module. In order to minimize delays caused by memory read/write operations, AXI-Stream and DMA are used for transmitting weight and feature map data. Given that the U-Net-light model consists of 30 layers and is fully implemented in hardware (PL-end), it consumes resources and has low reusability. Consequently, a neural network accelerator IP core has been developed to assist the ARM core processor in realizing the forward inference prediction process of the U-Net-light model. The accelerator primarily accelerates the convolution operation of each layer while maximum pooling and upsampling are computed by software on the PS terminal. The configuration of the accelerator (e.g., input/output image dimensions) is managed on the PS terminal.

### 4.2. Kernel: Accelerator

The findings from the C++ operations indicate that the convolution operation is the lengthiest and most prominent layer within the network structure. As a result, the focus of accelerator design is on expediting convolution operations. This paper’s designed convolutional accelerator has been tailored to accommodate a maximum input image size of 128 × 128, with a focus on enhancing operational efficiency and minimizing resource overhead.

**Figure 8:**
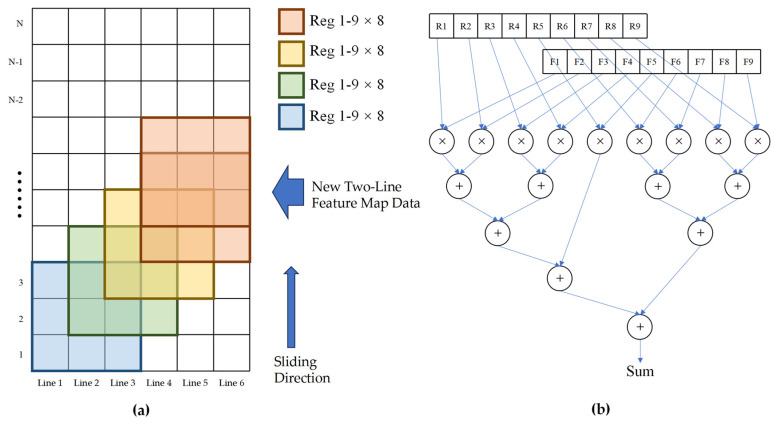
(**a**) Accelerator IP line buffer; (**b**) tree structure multiplication and accumulation.

The IP core is developed using HLS mode, with the addition of ap_fixed.h header file to specify the data type. The weight and feature map are set to 16-bit fixed-point numbers, with 6 bits for the integer part and 10 bits for the decimal part. To accommodate the size of the convolution kernel (3 × 3 pixels), 6 row buffers are set in BRAM, allowing for the simultaneous computation of 4 sets of convolutions. In the column dimension, columns are divided into 8 groups, each performing one convolution operation simultaneously. Within each group of clock cycles, a total of 32 convolution operations are calculated using a tree structure for multiplicative accumulation calculation as depicted in Figure 8b. After computing an image in the row buffer, it is shifted by two rows to prepare for the next round of computation. The implementation process of the IP core is illustrated in Figure 8a.

In the hardware implementation described above, the C code loop structure is configured using pragma, with the inner loop being expanded by unroll. The operation of the 3 × 3 convolution kernel is transformed into a combination of logic for multiplication and accumulation, allowing for the simultaneous calculation of 32 convolutions. During the convolution calculation process, it is necessary to read feature graph values and weight bias values from BRAM to registers, perform calculations, and then write back the results to BRAM. A three-level pipeline calculation is achieved using pipeline parameters. Bias value stacking is completed before writing back to BRAM at the end of the calculation. The row buffer array in BRAM has been unfolded using cyclic expansion mode partitioning and stored in 8 blocks, respectively, to increase memory read/write interfaces and resolve issues related to read/write conflicts and data dependency within the pipeline.

## 5. Results

### 5.1. Training Result

In this study, we utilized a self-constructed dataset to train a neural network for the purpose of the crown segmentation of Pinus tabuliformis and Salix matsudana. The training process was completed using GPU computing with NVIDIA GeForce RTX 3080 Ti. Detailed specifications are provided in Table 1.

The U-Net-light training utilizes the Adaptive Moment Estimation algorithm, with VGG16 serving as the feature extraction network. The model is built using the PyTorch framework within a Python virtual environment created in Anaconda3 with Python, and adapted to CUDA 11.0 for efficient processing. Detailed training setting parameters can be found in Table 2 of this paper.

**Figure 9:**
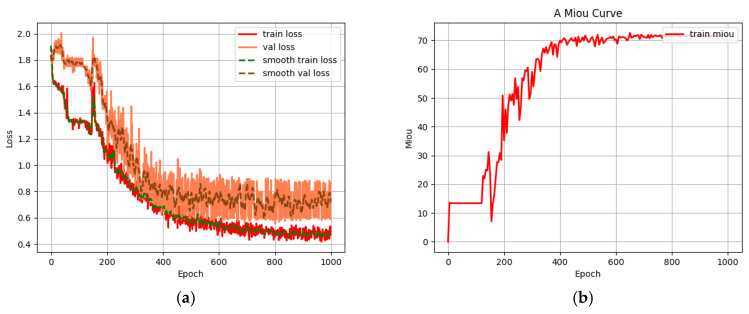
(**a**) Loss function of training set and verification set; (**b**) MIoU value curve during training.

Figure 9a presents the loss function values for both the training and validation sets of the U-Net-light model. The red solid line represents the training set loss, while the orange solid line denotes the validation set loss. Due to the noisy nature of the original data, both sets were smoothed and filtered to allow for easier interpretation. The green dotted line shows the smoothed loss curve for the training set, and the brown dashed line represents the smoothed loss curve for the validation set. As the number of iterations increases, both loss curves exhibit a consistent downward trend. Once the number of iterations reaches 800, the validation set loss stabilizes, indicating the successful convergence of the training model.

Figure 9b illustrates the MIoU value during the training process of the U-Net-light model. The graph shows that when the number of iterations reaches 800, the MIoU value stabilizes, further confirming that the model training is complete.

### 5.2. Segmentation Effect

**Figure 10:**
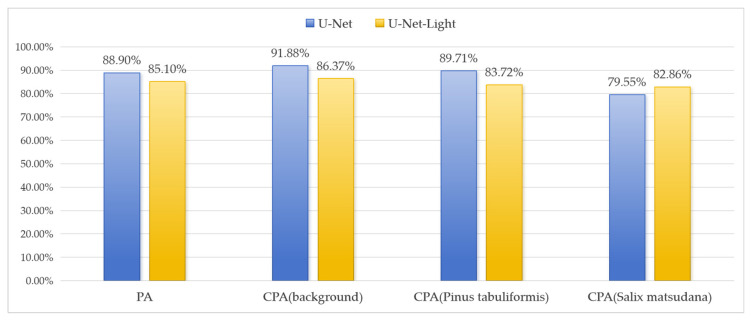
Accuracy comparison between U-Net and U-Net-Light.

**Figure 11:**
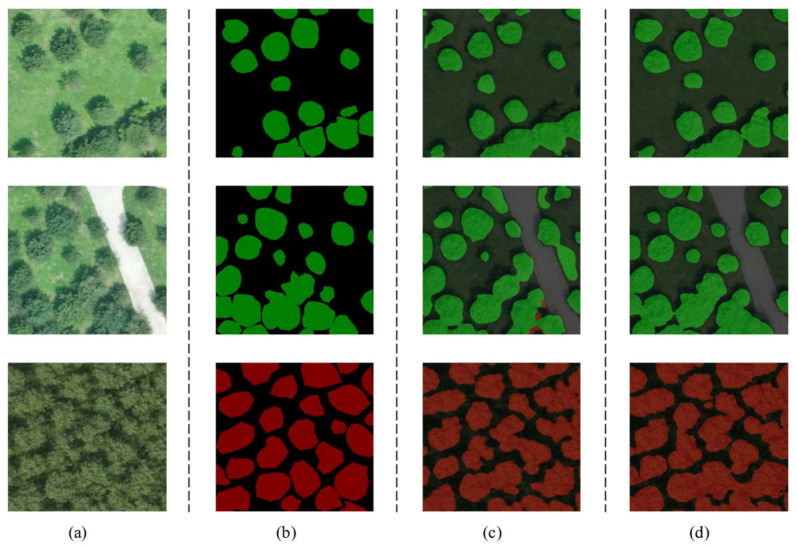
(**a**) Original images; (**b**) labeling result of the image; (**c**) U-Net-light segmentation result; (**d**) U-Net segmentation result (green area: Pinus tabuliformis; red area: Salix matsudana).

For the same test set images, predictions were made using both the U-Net-light and U-Net models, and the segmentation pixel accuracy (PA) and category pixel accuracy (CPA) were calculated for each test image. The result is shown in Figure 10. The average values were then used as quantitative indices for model evaluation. The average PA for U-Net-light segmentation is 85.1%, while the average PA for U-Net segmentation is 88.9%, indicating that both models were successfully constructed and trained. U-Net-light achieves only a 3.8% decrease in PA despite a significant reduction in parameters, demonstrating the effectiveness of the model’s lightweight design. For the U-Net-light model, the average CPA for background segmentation is 86.37%, for crown segmentation is 83.72%, and for salix tree crown segmentation is 82.86%. In comparison, the U-Net model achieved an average CPA of 91.88% for background segmentation, 89.71% for crown segmentation, and 79.55% for salix tree crown segmentation. The performance difference between the U-Net-light and U-Net models ranges from 3% to 5%, with U-Net-light even outperforming U-Net in certain aspects, such as salix tree crown segmentation. The observed phenomenon may be attributed to the inherently complex canopy structures of Salix matsudana, where the lightweight architecture of U-Net-Light effectively mitigates overfitting risks inherent in deeper networks like U-Net. These results validate the effectiveness of the innovative U-Net-light network structure proposed in this study. Figure 11 presents selected segmentation examples, including three types of remote sensing images: Pinus tabuliformis forest, Pinus tabuliformis forest with a road background, and Salix matsudana forest. The segmentation results are shown to be satisfactory.

### 5.3. Run Time

In this study group, guest ZYNQ7100 SoC designs are verified on the FPGA development board and tested to verify the forward reasoning results and test the acceleration of the SoC effect in order to reflect the rationality of the design of SoC, and at the same time in 20 core cpus (Intel CoreTM i9 CPU-10900 k, The U-Net-light software (Python: 3.8.8, PyTorch: 1.7.1, CUDA: 11.0, ONNX: 1.12.0) calculation was implemented on the 3.70 GHz and ARM single-core (Cortex-A9, 800 MHz) as a reference.

**Figure 12:**
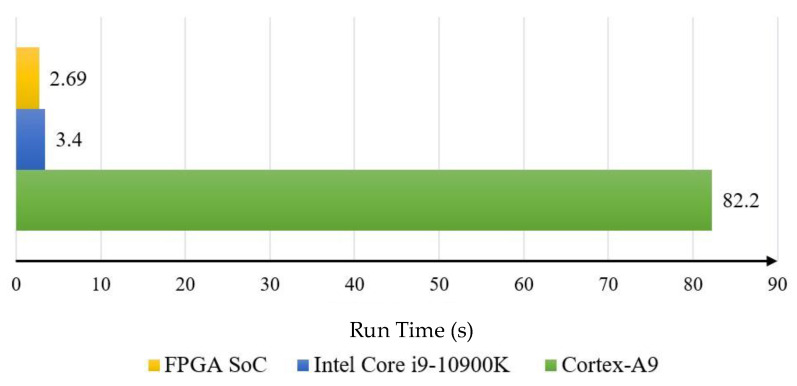
Total running time of U-Net-light on different platforms.

In this study, five rounds of tests were conducted to measure the inference time required for processing 128 × 128-pixel images. The data in Table 4 represent the average performance across multiple tests using the same input image to ensure consistency. Table 4 details the time taken to run each layer of U-Net-light on three different platforms, while Figure 12 illustrates the total runtime. The results show that the shortest runtime was 2.69 s on the FPGA compared to 3.4 s on the Intel Core i9-10900K CPU and 82.2 s on the Cortex-A9 ARM embedded core. The accelerator IP core designed by this research achieved a 31-fold acceleration compared to the ZYNQ PS end core, and the SoC architecture provided a 1.3-fold acceleration compared to the high-end CPU (20-core i9-10900K). These results demonstrate the effectiveness and rationality of the accelerator IP and SoC architecture design, successfully achieving neural network acceleration.

## 6. Discussion

### 6.1. Comparison of U-Net and U-Net-Light Models

As shown in Table 5, for images with the same input size (512 × 512 pixels), the U-Net-light model achieves a 93.7% reduction in both the number of parameters and the computational load compared to the U-Net model. With only 1.56 MB parameters, U-Net-light is an exceptionally lightweight network, making it suitable for deployment in edge computing scenarios such as embedded terminals. The slight reduction in accuracy—within 5%—is offset by a more than 90% improvement in model efficiency and speed, further validating the rationality and necessity of the innovative network structure developed in this study.

**Figure 13:**
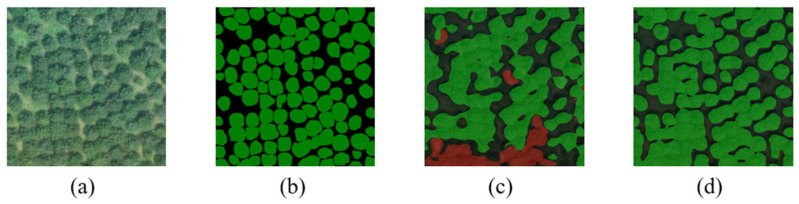
(**a**) Original images; (**b**) labeling result of the image; (**c**) U-Net-light segmentation error; (**d**) U-Net segmentation result (green area: Pinus tabuliformis; red area: Salix matsudana).

When the U-Net-light model is applied to image scaling and other transformations, its segmentation accuracy is slightly lower than that of the U-Net model, with an average segmentation PA of only 60.9% compared to 85.0% for U-Net. This indicates that the U-Net-light model has weaker generalization ability, highlighting an area for further improvement. An example of this error is shown in Figure 13.

### 6.2. FPGA Resource Overhead and Energy Consumption

#### 6.2.1. SoC Hardware Resource Overhead

After synthesis, the estimated resource costs for the SoC chip can be observed in Vitis HLS, as shown in Table 6. In this study, the implementation of the neural network accelerator IP core occupies a moderate amount of resources, with the DSP component, which consumes the most resources, utilizing only 27%. The remaining logic units, lookup tables, flip-flops, and Block RAM account for just 2% to 3% each. The accelerator IP core was adapted to the selected Milunk-ZYNQ-MZ7100 development board and validated on the ZYNQ programmable SoC platform.

**Figure 14:**
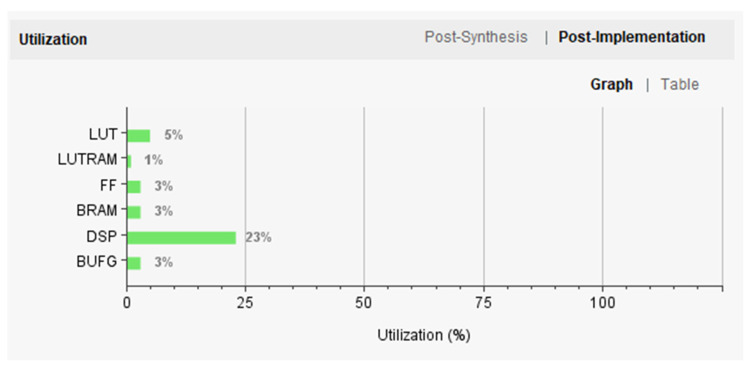
Overall ZYNQ 7100 SoC utilization.

After implementing all the SoC designs on the ZYNQ-MZ7100 development board, the overall resource utilization is depicted in Figure 14. Through the subsequent optimization of layout and routing, resource utilization was further improved. Specifically, DSP utilization decreased to 23%, while the usage of lookup tables, which is higher than logical synthesis, reached 6%. BRAM utilization saw a slight increase of 1%. The optimization resulted in a balanced use of various resources. The SoC architecture designed in this study utilizes less than a quarter of the hardware resources of the FPGA platform, demonstrating its potential for migration to other Xilinx FPGA series. This indicates that the SoC architecture design and IP development in this study have significant theoretical and practical value.

#### 6.2.2. Energy Consumption

**Figure 15:**
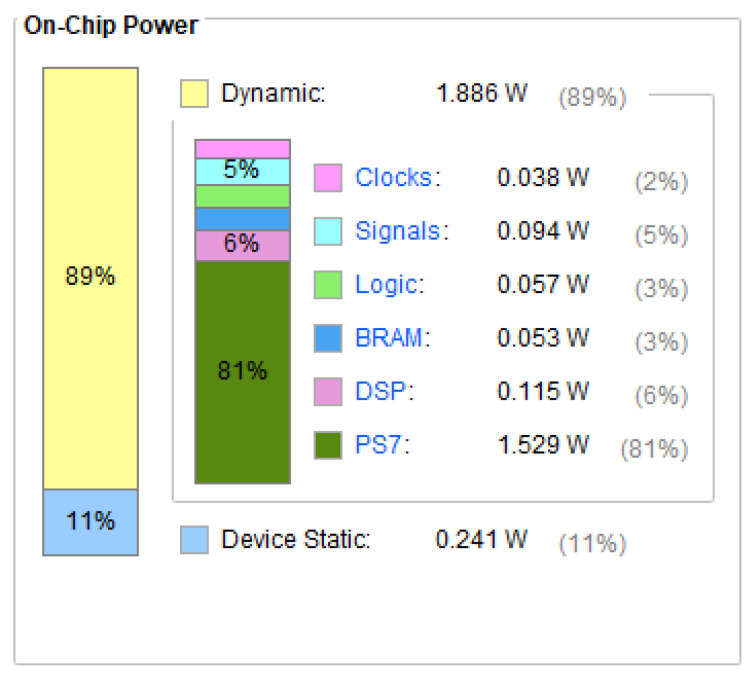
Zynq 7100 on-chip power report.

Figure 15 shows the on-chip power consumption of the SoC designed in this study. Although this design has some latency limitations compared to high-performance graphics processors such as the NVIDIA GeForce RTX 3080 Ti GPU, it excels in energy efficiency. The on-chip power consumption is only 2.127 W, with static power consumption at 0.241 W and dynamic power consumption at 1.886 W. In contrast, the NVIDIA GeForce RTX 3080 Ti GPU consumes up to 350 W, the Intel Core™ i9-10900K CPU uses 251.6 W, and even high battery life notebooks consume over 15 W. Energy-saving lamps typically consume no less than 3 W. This demonstrates that the SoC designed in this study achieves a significant reduction in power consumption.

### 6.3. Practical Implications and Challenges in Real-World Applications

The proposed U-Net-light model and FPGA-based SoC architecture hold significant potential for real-world applications beyond the studied forest farm. For instance, in environmental conservation, this system could monitor deforestation or track biodiversity changes by segmenting tree crowns in protected areas. In precision agriculture, it could assess interspersed crop health alongside tree vitality, offering a dual-purpose solution for agroforestry management. Urban planners might leverage this technology to evaluate green space distribution in cities, ensuring compliance with sustainability goals.

However, deploying this method in diverse environments presents challenges. Variability in tree species, particularly in tropical or mixed forests with overlapping canopies, may reduce segmentation accuracy. Seasonal changes, such as leaf shedding in deciduous trees, could further complicate model performance. Additionally, UAV-based data collection is sensitive to weather conditions (e.g., fog and rain), which may necessitate robust preprocessing pipelines or adaptive algorithms. Scalability is another concern—processing high-resolution imagery across vast forested regions demands optimized data transmission and storage strategies, especially in remote areas with limited connectivity.

## 7. Conclusions

This paper focuses on the Jingyue Ecological Forest Plantation in Changping District, Beijing, and presents the design and training of a lightweight convolutional neural network, U-Net-light, tailored for embedded platforms and FPGAs. Based on the U-Net and VGG16 models, U-Net-light is optimized for crown width segmentation in mixed forests with a parameter count of just 1.56 MB. The model successfully classifies trees and segments crown widths for Pinus tabuliformis and Salix matsudana, achieving a reasoning accuracy of 85%. Deployed on the Xilinx ZYNQ 7100 programmable SoC platform, the U-Net-light model processes 128 × 128 pixel RGB tree crown images in just 2.69 s. The accelerator IP core designed in this study delivers a 31-fold acceleration compared to the ZYNQ PS end core, and the SoC architecture achieves a 1.3-fold acceleration compared to the high-end Intel Core™ i9-10900K CPU. In terms of hardware resource usage, the system consumes less than one-quarter of the target platform’s resources, with a total on-chip power consumption of only 2.127 W.

This paper proposes a lightweight deep learning model, U-Net-light, for tree canopy segmentation and tree species recognition. The study presents an SoC architecture and neural network accelerator IP core that enable the deployment of the U-Net-light model, achieving notable acceleration and demonstrating high platform versatility. In conclusion, this study holds significant theoretical value and promising practical applications.

The U-Net-light model and its FPGA-based SoC implementation exhibit certain limitations that require further refinement. The model’s generalization capability remains insufficient, evidenced by reduced pixel accuracy when handling image scaling and transformations, which limits its robustness in diverse forest environments such as complex canopy structures, varying light conditions, or seasonal changes. Additionally, performance degradation occurs in high-density canopy segmentation, making it challenging to accurately identify individual tree canopies in tropical or mixed forests. The dependence on manual annotations for training hinders scalability across large datasets. On the hardware side, while HLS development methods improve development efficiency, they deliver lower real-time performance compared to Verilog and other HDLs, especially with complex models. Current accelerators are optimized for convolution operations but lack adaptability to diverse network architectures, and system scalability is limited by data transmission bottlenecks, storage constraints, and environmental factors like weather conditions. Future improvements could focus on: optimizing accelerator designs for complex models, exploring hybrid HLS-HDL approaches, introducing data augmentation and semi-supervised learning to enhance generalization and reduce annotation dependency, and improving on-chip memory management to address environmental challenges.

This study provides theoretical methods and practical cases for the integration of lightweight deep learning models with edge computing. Its characteristics of low power consumption and high energy efficiency ratio have direct application value in fields such as dynamic forest resource monitoring and smart agriculture. In the future, through interdisciplinary collaboration and hardware iteration, this technology can be further advanced from laboratory validation to engineering implementation.

## Figures and Tables

**Table 1:** Specifications of NVIDIA GeForce RTX 3080 Ti GPU.

Specification	Value
Number of NVIDIA CUDA Core	10,240
Accelerating Frequency	1.67 GHz
Base frequency	1.37 GHz
Video memory capacity	12 GB
Video memory type	GDDR6X
Max GPU temperature (°C)	93
Video Card Power (W)	350

**Table 2:** Parameters in training U-Net-light network based on PyTorch.

Parameters	Value
Training picture size	512 pixels × 512 pixels
Sort quantity	3 (2 tree species + background)
Iteration round	1000
Lot size	2
Maximum learning rate	0.0001
Minimum learning rate	0.000001
optimizer	Adam
Momentum of the optimizer	0.9
weight decay	0

**Table 3:** Main parameters of ZYNQ-MZ7100 development board.

Parameter	Value
PS DDR	DDR3L 1GB 1066 MHZ × 32 bits
PS XTL	33.3333 MHZ
PL IO Count	207
Core Power	1.0 V power supply, maximum output 30 A
USB Serial Interface	Supports PS UART
SD Card Slot	Supports MicroSD card
HDMI Interface	Bidirectional input/output

**Table 4:** Running time of each layer of U-Net-light on different platforms (unit: ms).

Network Structure	Cortex-A9	Intel Core i9	FPGA SoC
conv 1-1 + relu	680.500	30.263	29.365
conv 1-2 + relu	3499.831	144.641	96.791
maxpool 1	17.540	0.9985	17.540
conv 2-1 + relu	1747.908	71.2556	49.648
conv 2-2 + relu	3480.029	143.154	92.371
maxpool 2	8.539	0.4	8.539
conv 3-1 + relu	1738.230	70.49	48.797
conv 3-2 + relu	3472.514	144.302	94.109
conv 3-3 + relu	3472.608	144.063	94.110
maxpool 3	4.297	0.2149	4.297
conv 4-1 + relu	1733.709	71.2383	52.764
conv 4-2 + relu	3470.381	143.322	103.760
conv 4-3 + relu	3470.328	143.337	103.759
maxpool 4	2.215	0.1043	2.215
conv 5-1 + relu	867.844	35.5283	32.654
conv 5-2 + relu	867.823	36.0041	32.651
conv 5-3 + relu	867.832	35.975	32.649
upsamping 1	12.915	0.6204	12.915
conv 6-1 + relu	6989.754	291.702	205.750
conv 6-2 + relu	3470.225	143.191	103.761
upsamping 2	52.141	2.5041	52.141
conv 7-1 + relu	10,455.390	442.901	275.357
conv 7-2 + relu	3472.430	143.66	94.109
upsamping 3	104.801	5.247	104.801
conv 8-1 + relu	10,433.333	436.347	263.267
conv 8-2 + relu	3479.452	138.293	92.371
upsamping 3	210.146	11.1664	210.146
conv 9-1 + relu	10,446.885	428.636	262.764
conv 9-2 + relu	3500.994	141.288	96.791
conv 10	78.393	3.9164	18.150
Total Time	82,200	3400	2690

**Table 5:** Comparison of parameters and computation between U-Net and U-Net-light.

	U-Net	U-Net-Light
Total GFLOPS	451.672 G	28.663 G
Total params	24.891 M	1.557 M

**Table 6:** Usage of IP hardware resources of neural network accelerator.

Name	BRAM_18K	DSP	FF	LUT	URAM
DSP	-	552	-	-	-
Expression	-	-	0	9807	-
FIFO	-	-	-	-	-
Instance	0	4	138	253	-
Memory	42	-	768	96	-
Multiplexer	-	-	-	738	-
Register	-	-	10,349	32	-
Total	42	556	111,255	10,926	0
Available	1510	2020	5,554,800	277,400	0
Utilization (%)	2	27	2	3	0

## Data Availability

The datasets used and/or analyzed during the current study are available from the corresponding author upon reasonable request.
